# An atypical presentation of gastrointestinal stromal tumour: a case report

**DOI:** 10.1093/jscr/rjac471

**Published:** 2022-10-30

**Authors:** Fahreyar Alam, Matthew How Saw Keng, Harry R Haynes, Filip Tsvetkov, Mohamed Tourky, Richard Payne

**Affiliations:** Department of General Surgery, Great Western Hospital NHS Trust, Swindon, UK; Department of General Surgery, Great Western Hospital NHS Trust, Swindon, UK; Department of General Surgery, Great Western Hospital NHS Trust, Swindon, UK; Department of General Surgery, Great Western Hospital NHS Trust, Swindon, UK; Department of General Surgery, Great Western Hospital NHS Trust, Swindon, UK; Department of General Surgery, Great Western Hospital NHS Trust, Swindon, UK

**Keywords:** GIST, myelo-proliferative disorder, anaemia

## Abstract

Gastrointestinal stromal tumours (GIST) occur more commonly in the stomach and make up ~80% of the GI mesenchymal neoplasms. They are very rare in young adults and in males. The diagnosis is confirmed histologically and immunohistochemically. Once diagnosed, survival rates are dependent on various factors. The main treatment is resection, but targeted therapy can be used pre or post-operatively. This case is of a 35-year-old female with no significant medical history presenting to her general practitioner with lethargy, malaise and mild weight loss. Initially, she was investigated for a haematological malignancy, but upon further investigations, her computed tomography (CT)  scan showed an abdominal mass, which was resected and found to be a high-grade perforated gastrointestinal stromal tumour in her proximal ileum. This is a good example of an atypical presentation of GIST and emphasizes the importance of thorough workup and prompt surgical intervention in achieving a satisfactory outcome.

## INTRODUCTION

Gastrointestinal stromal tumours (GIST) occur in the gastrointestinal (GI) tract due to proliferation of the GI mesenchymal cells. This is due to the abnormal form of tyrosine protein kinase (KIT) known as CD117. KIT up regulates stem cell precursors of the interstitial cells of Cajal, resulting in tumour formation. The tumours originate anywhere from the oesophagus to the anus [1]. The most common site is the stomach (40–70%) followed by small intestine (15–44%) and is rarely found on the omentum, mesentery and retroperitonium (2–11%)[2]. They make up 80% of the GI mesenchymal neoplasms [3] that are more commonly seen in those around 60 years of age [2]. They are rare in children and young adults [4], and prevalence is higher in males than females [2].

Patients are usually asymptomatic and the GIST is found incidentally or on exploration [3]. The severity of symptoms is dependent on the size and site. Large tumours present with abdominal pain, indigestion and early satiety [2].

GI bleeding can occur in half of the cases and presents with symptoms of anaemia, melaena or haematemesis. GI bleeding and abdominal pain are more commonly associated with gastric GIST, whereas acute abdominal symptoms are associated with jejunal/ileal GISTs [5]. Rupture of GIST tumours is a rare complication and is reported as 8% of GIST-related emergencies [6].

Computed tomography (CT) is the gold standard modality for determining the size, location and spread. Positron emission tomography can also be useful in the localization of secondary lesions [2]. However, diagnosis is made histologically and immunohistochemically. This allows for differentiating from other mesenchymal tumours [7]. Immunohistochemically, the diagnosis of GISTs are made with CD117 positivity [8]. According to the National Comprehensive Cancer Network, CD117 is positive in 95% of the cases. Once diagnosis is made, prognostic factors include size, site and mitotic index. GISTs > 5 cm have a high risk of recurrence and GISTs localized to the stomach have a better survival rate [9]. There are many risk stratifications systems for GIST.

The goal standard treatment is surgical resection. In patients with locally advanced tumours or metastasis, adjuvant targeted therapy using tyrosine kinase inhibitors (Imatinib) can be used [2].

This case report presents an atypical presentation of GIST in a young, previously well female who presented with a general feeling of fatigue, lower abdominal pain with anaemia, thrombocytosis and lymphocytosis. CT of abdomen and pelvis showed a small bowl mass for which she underwent a semiurgent laparotomy (small bowl resection and primary anastomosis). The post-operative histology later confirmed a perforated GIST, which is a rare complication of GIST [6].

## CASE REPORT

A 35-year-old female presented to the hospital with lethargy, exertional dyspnoea, anorexia and weight loss. This was accompanied by vague abdominal symptoms and lower abdominal discomfort. One week prior, she reported of having fever and supra pubic pain, which was treated for a suspected urinary tract infection.

Her medical history included cobalamin deficiency and seasonal allergies. Drug history included four monthly B12 injections. She was a non-smoker and her alcohol consumption was reported minimal. She was independent with her daily activities.

Her routine blood tests showed that she had a normocytic anaemia (Hb = 70 g/l). Her iron studies were normal, B12 was high and folic acid was normal.

She had a raised white blood cell count (17 × 10^9^ cells/l), platelets (1183 × 10^9^ cells/l) and C reactive protein of 108 mg/l. She was subsequently referred to the haematology department for a suspected myeloproliferative disorder.

**Figure 1:**
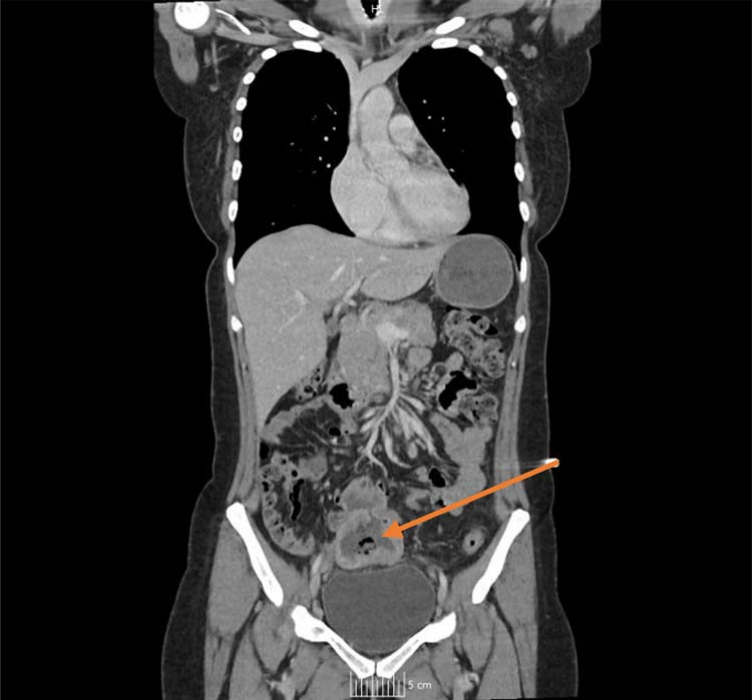
CT coronal image, with arrow showing the midline lower abdominal/pelvic thick walled fluid and gas-filled structure that measured 5 × 7 × 6 cm.

**Figure 2:**
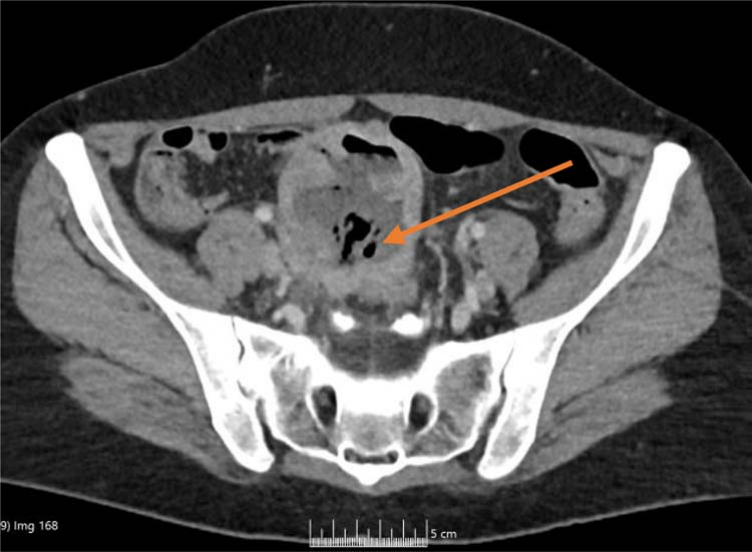
CT axial image, with arrow showing the midline lower abdominal/pelvic thick-walled fluid and gas-filled structure that measured 5 × 7 × 6 cm.

Once admitted to the haematology department, she was examined and was found to have a soft, non-tender abdomen with no signs of peritonism, and all her other systemic examinations were normal. Given the vague abdominal symptoms and blood profile, she was subjected to a CT scan of her chest, abdomen and pelvis (CT CAP). The scan reported a lower abdominal/pelvic thick-walled fluid and gas-filled structure that measured 5 × 7 × 6 cm lying adjacent to the urinary bladder with a likely fistulous tract to the overlying loops of bowel. A small amount of free fluid and non-specific stranding of fat was seen in the pelvis. No pulmonary or metastatic nodules were seen (Figs 1 and 2).

The haematology team subsequently referred her to the general surgeons. Surgical review revealed a pale patient, with sinus tachycardia of 94/min. Remaining observations were unremarkable. Abdominal examination revealed a non-distended, soft, mildly tender lower abdomen with no visceromegaly. Keeping in view of the history, clinical findings, blood picture and the results of the CT CAP, she was teed up for a semiurgent laparotomy. Differential diagnosis included Meckel’s diverticulum, tubo-ovarion pathology and small bowl lymphoma. Her preoperative morbidity and mortality according to National Emergency Laparotomy Audit score was 10 and 1.5%, respectively.

Laparotomy revealed localized pelvic sepsis, a large perforated inflammatory mass arising from the proximal ileum firmly adherent to the dome of the urinary bladder and the sigmoid colon. Because of the significant inflammatory fibrosis, with no planes of dissection, the small bowl mass was mobilized off the urinary bladder and the sigmoid colon via blunt dissection. The mass was resected via a small bowl resection and a primary hand-sewn, double-layered anastomosis was fashioned. Thorough lavage of the peritoneal cavity was performed with normal saline. Specimen was sent for histology and immunohistochemistry.

**Figure 3:**
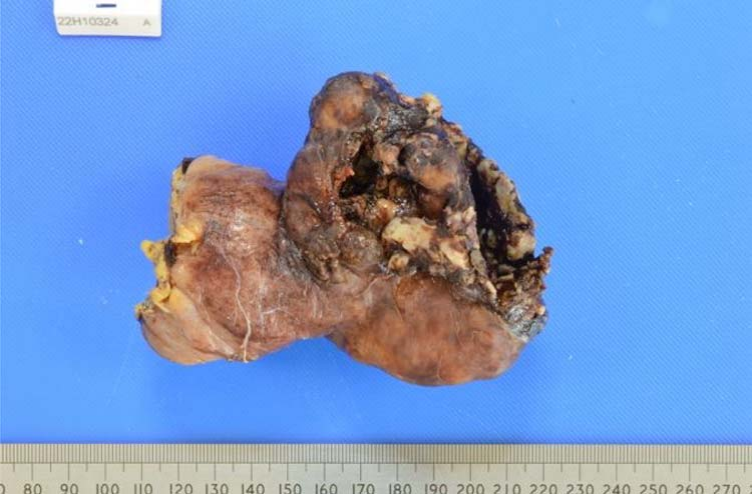
Macroscopic view of the ruptured GIST.

The patient made an uneventful post-operative recovery with no residual symptoms. The histology came back reporting a ruptured GIST measuring 60 × 70 × 60 mm in size with a 3a prognostic GIST category according to the AFIP risk stratification scheme (Fig. 3).

**Figure 4:**
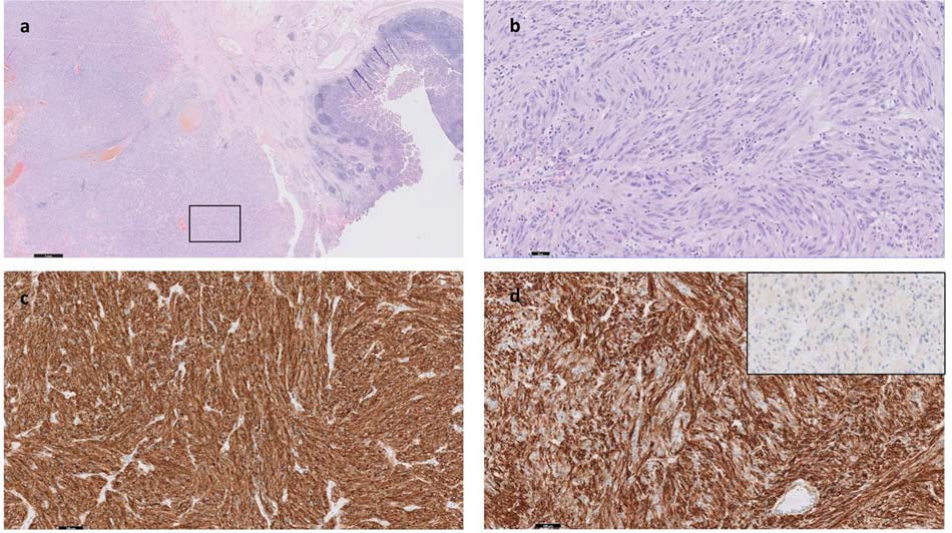
The malignancy consists of diffuse sheets and fascicles of pleomorphic and atypical cells with nuclear hyperchromasia and irregular nuclear membranes; there are focal intra-nuclear inclusions; The cells have fairly abundant eosinophilia cytoplasm; <5 mitoses per 5 mm^2^; CD117 (KIT) IHC is positive (**c**). CD34 IHC positive **(d)**. MelanA is negative (inset, d). DOG1, Desmin and PanCK are negative (not shown). The boxed area in **(a)** is shown in high magnification in **(b)**.

Immunohistochemistry showed diffused CD117 positivity and subtotal immunopositivity of CD34 and SMA (Fig. 4).

The patient was then subsequently referred to a tertiary centre multi-disciplinary team meeting for further management.

## CONCLUSION

GISTs can present in an atypical fashion and can be difficult to diagnose. Clinicians should be aware of the latter. This case report was a good example of an atypical presentation of GIST, which was initially thought to be a haematological malignancy. However, a good outcome can be achieved through thorough investigations and prompt surgical intervention.
